# Effects of moisture content on temperature stability and microbial population growth over time in a dry cow total mixed ration

**DOI:** 10.3168/jdsc.2025-0978

**Published:** 2026-03-27

**Authors:** L. Garcia, F.F. Cardoso, F.C. Cardoso

**Affiliations:** 1 Department of Animal Sciences, University of Illinois, Urbana, IL 61801; 2 Department of Animal & Avian Sciences, University of Maryland, College Park, MD 20742

## Abstract

Controlling energy with high-fiber diets throughout the entire dry period is a nutritional strategy aimed at preventing overconsumption of nutrients, but this may result in lower moisture content. Conversely, TMR with high moisture content are more prone to spoilage, especially under elevated environmental temperatures. High temperatures signal spoilage caused by microorganisms present in the TMR, which generate heat and diminish the ration's nutrient value. Therefore, the objective of this study was to evaluate the effects of the addition of water to a dry cow TMR containing wheat straw (37% of total dietary DM) on TMR temperature and microbial populations. Three separate runs were conducted, each with six 3.78-L buckets randomly allocated to 2 treatment groups (n = 3 per treatment): NW (with no water added; 49% DM) and WW (with 7.3 kg of water added to the mixture; 43% DM). Temperature recording began immediately after filling the buckets with their corresponding treatments using data loggers (n = 3 per bucket; Thermochron iButtons, iButtonLink, Whitewater, WI), placed at the bottom, middle, and top of each bucket. The average temperature for all locations was used. Data loggers were set to record at 5-min intervals and summarized as hourly averages for 24 h. In addition, data loggers (n = 3) were placed outside of the buckets to register ambient temperature. Samples of TMR were collected at 0, 6, 12, 18, and 24 h after the mixture was prepared and placed into buckets. Three samples per treatment were composited at each time point and the composites were plated for yeast, total bacteria, and clostridial counts. Data were analyzed using the MIXED procedure in SAS (SAS Institute Inc.). There was no treatment effect for TMR temperature (NW = 29.1°C ± 0.76°C and WW = 30.6°C ± 0.76°C). A main effect of time point (h) was observed for TMR temperature, total bacteria, and yeast. The TMR temperature tended to be higher for WW (34°C ± 1.56°C, 35°C ± 1.59°C, 35°C ± 1.57°C, 35°C ± 1.49°C) than NW (29°C ± 1.56°C, 29°C ± 1.59°C, 30°C ± 1.57°C, 30°C ± 1.49°C) at 16, 17, 18, and 19 h, respectively. In contrast, NW had increased yeast counts compared with WW. Overall, water addition to a dry cow TMR appeared to affect feed temperature after 16 h of its preparation and reduced yeast growth over time but did not affect total bacterial or clostridial counts.

## Highlights

• Yeast populations were reduced by 30% with water added to a high-straw dry cow TMR compared with no water added to the TMR.
• Addition of water in a high-straw dry cow TMR increased TMR temperature by, on average, 17.8% from 16 to 19 hours compared with no water inclusion.
• The progressive increases in TMR temperature and microbial populations over time imply that, during warmer weather, these results would be more relevant than in cooler conditions.

## Graphical Abstract

**Figure:**
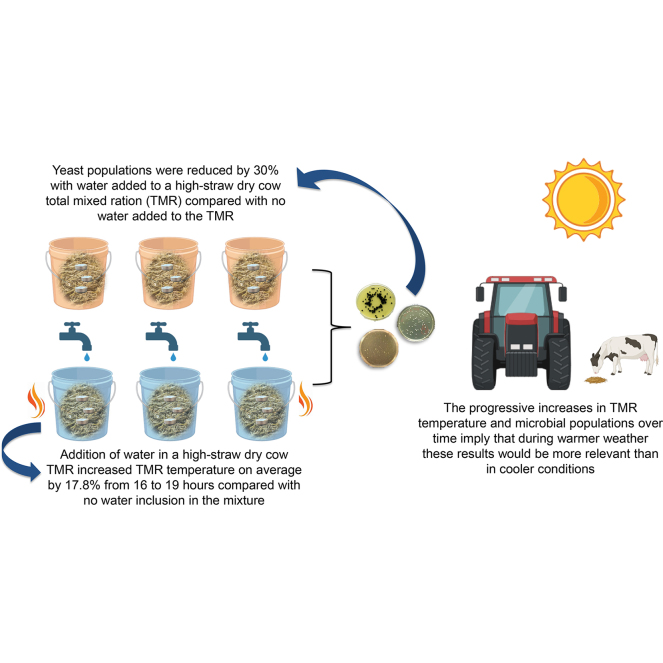
**Summary:**During the prepartum period, the energy content of diets is often diluted with less nutrient-dense feedstuffs to prevent dairy cows from overconsuming nutrient requirements, resulting in a lower moisture dry cow ration. Water is commonly added to the ration, but this raises the risk of feed spoilage. Our objective was to evaluate the effects of water addition on total mixed ration (TMR) temperature and microbial populations in a dry cow diet containing wheat straw. We concluded that adding water to a dry cow diet increased TMR temperature at 16 to 19 hours after feeding compared with no water addition; however, yeast counts were greater when water was not included in the mixture. Total bacterial populations increased over time, and clostridial growth tended to increase as hours after feeding progressed. Some images created with BioRender.com.

Prepartum dairy cows, specifically those in late gestation, are managed to maintain a consistent DMI of a properly balanced diet before calving to promote greater metabolic health postcalving (Goldhawk et al., 2009). A nutritional strategy used during the dry period to ensure consistent DMI is supplying diets with a greater inclusion of less nutrient-dense feedstuffs to increase NDF content and limit voluntary DMI (NRC, 2001), thereby restricting total nutrient consumption so that cows do not exceed their daily energy requirements. Drackley and Janovick Guretzky (2007) recommended incorporating cereal straws, specifically wheat straw prechopped to ≤2 in in length, to dilute the energy content of diets containing higher-energy ingredients. Controlling energy with high-fiber diets throughout the entire dry period has been reported to improve DMI after calving (Douglas et al., 2006), whereas postpartum concentrations of BHB and nonesterified fatty acids were reduced (Mann et al., 2015; Richards et al., 2020). Because a high-fiber dry cow TMR typically contains less moisture than a lactating TMR, it has been suggested that adjusting the moisture content to more closely align to that of the lactating TMR may help ease the cow's transition to physiologically adapt to dietary changes (Havekes et al., 2020). As such, incorporating water into the dry cow TMR has traditionally been used to better manage the amount of feed sorting (Shaver, 2002). Cows fed a high-straw dry cow diet with 53.4% DM had reduced DMI during the dry period and tended to sort more in favor of medium particles (<19 mm, >8 mm) than cows fed a high-straw diet with 45.4% DM (Havekes et al., 2020). However, TMR with high moisture content are known to spoil more frequently under elevated environmental temperatures (Eastridge, 2006) and prone to heating (Felton and DeVries, 2010). Under aerobic conditions, yeast metabolize lactic acid, which leads to oxidation of nutrients and subsequent heating of the silage. Spoiled silage is unfavorable because the nutritive value is diminished (Kung et al., 2018), and it has been hypothesized that spoilage in feed bunks can further lead to reduced DMI and greater sorting (Felton and DeVries, 2010). Previous research reported that a greater inclusion of water in a lactating TMR resulted in greater increases in feed temperature in the hours after feed delivery, greater sorting against long particles, and reduced DMI (Felton and DeVries, 2010), suggesting feed spoilage may have occurred. Tools to identify such spoilage through changes in feed characteristics include monitoring temperature, pH, and yeast counts (Kung et al., 1998). Further investigation is needed to identify microorganisms contributing to spoilage in the TMR and to determine the time points at which their activity becomes significant. Therefore, the objective of this study was to evaluate the effects of water inclusion on TMR temperature and microbial populations in a dry cow diet with the inclusion of wheat straw (37% of total dietary DM). We hypothesized that the inclusion of water in a high-straw dry cow TMR would increase the TMR temperature and alter microbial populations compared with a dry cow TMR without water inclusion.

A total of six 3.78-L size buckets were randomly allocated into 2 treatment groups: **NW** (dry cow TMR with no water added; 49% DM) and **WW** (dry cow TMR with 7.3 kg of water added to the mixture; 43% DM). The experimental period was conducted from August to September 2021 and assembled in 3 separate runs. Each experimental run included all 6 buckets (n = 3 per treatment). The dry cow TMR was mixed at approximately 1230 h using a Keenan Mech-Fiber 320 mixer (Alltech Farming Solutions Limited, Borris, Co. Carlow, Ireland). Forages were loaded first into the mixer, followed by the concentrate ingredients. For WW, water was loaded into the mixer as the last ingredient. The TMR was mixed for 3 min at 390 rpm and delivered to the barn at approximately 1300 h. We determined the temperature stability of each treatment by placing approximately 4 kg of TMR in clean buckets and measuring the temperature every 5 min for a total of 24 h using data loggers (n = 3 per bucket; Thermochron iButtons, iButtonLink, Whitewater, WI). Buckets were placed adjacent to one another by treatment inside an enclosed barn and were left uncovered during the incubation, with no additional insulation provided. Additional calculations were made to ensure that the experimental design had the power to detect a minimum of 1% for TMR temperature, assuming a power of 0.8 and a 2-tailed α of 0.05. To measure TMR temperature, data loggers were placed at the bottom, middle, and top of each bucket, and the hourly average temperature for all locations was used. Separate data loggers were positioned outside and adjacent to the buckets to register ambient temperature. In addition, barn temperature and humidity were monitored at 15-min intervals using a HOBO Pro logger (Onset Computer Corp., Pocasset, MA). Temperature-humidity index (**THI**) was calculated using the equation of McDowell et al. (1976). To conduct microbial counts, treatment samples were collected at 0, 6, 12, 18, and 24 h after each treatment was mixed and placed into buckets. At each time point, one sample was collected from each bucket (n = 3 per treatment) and combined to form a single composite sample for each treatment. The composites were plated for yeast, total bacteria, and clostridial counts. For each microbial analysis, 22 g of the treatment samples were transferred into sterile homogenization bags, suspended 1:10 (wt/vol) in a sterile peptone solution, and manually masticated for 1 min. Yeast populations were enumerated through serial dilutions using potato dextrose agar (39 g/L) supplemented with tartaric acid (16 mL/L) and incubated at room temperature (25°C to 28°C) for 5 d. Total bacteria populations were enumerated through serial dilutions using tryptic soy agar (40 g/L) and incubated at 37°C for 24 h. Clostridia were enumerated through serial dilutions using tryptose sulfite cycloserine agar (41 g/L) supplemented with sterilized D-cycloserine solution (3.6 mL/L) and incubated at 37°C for 24 h inside an anaerobic container. Additionally, all microbial analyses used the pour plate technique for microbial enumeration and were performed in duplicate. Duplicates were averaged and means were considered as observations in the statistical analysis. To ensure that the water source was free of microbial contamination, water was plated, and no colonies were detected. The physical characteristics of the treatments, based on the Penn State Particle Separator (Kononoff et al., 2003), were assessed using upper (19-mm pore size), middle (8-mm pore size), and lower (4-mm pore size) sieves, plus the pan, and each treatment was evaluated in triplicate.

The statistical analysis was performed using SAS (v9.4, SAS Institute Inc., Cary, NC). For TMR temperature measured, the MIXED procedure of SAS was used to model the main fixed effect of treatment, time, and their interaction. The following model was used:*Y_ijkl_* = *µ* + *T_i_* + *H_j_* + (*T_i_* × *H_j_*) + *B_k_*_(_*_l_*_)_ + *ε_ijkl_*,where *Y_ijkl_* = the observations for dependent variables; *µ* = the overall mean; *T_i_* = the fixed effect of the *i*th treatment; *H_j_* = the repeated measurement effect of the *j*th time; *T_i_* × *H_j_* = the interaction between the treatment and time; *B_k_*_(_*_l_*_)_ = the random effect of bucket nested within run; and *ε_ijkl_* = the random residual error. Bucket was considered the experimental unit. For microbiology variables (i.e., clostridia, total bacteria, and yeast counts), statistical analysis was performed by including the fixed effects of treatments, time [T (time point)], and treatment × T interaction. Run was the experimental unit, included as a random effect and specified as the subject for the repeated measures, with time point as the repeated term. Variables were subjected to 5 covariance structures: compound symmetry, autoregressive order 1, autoregressive heterogeneous order 1, unstructured, and Toeplitz. Repeated measures in both analyses were analyzed using compound symmetry covariance structure that yielded the lowest corrected Akaike information criterion to account for constant correlation among observations within the experimental unit. The Kenward–Roger degrees of freedom estimation was used to determine the denominator degrees of freedom (Littell, 2002). Residuals distributions were assessed for normality and homoscedasticity. A quantile-quantile plot and box plot were used to determine outliers calculated by multiplying the interquartile range by 3 and subtracting or adding from the corresponding quartile. Statistical significance was considered at *P* ≤ 0.05 and tendencies at 0.05 < *P* ≤ 0.10.

The ingredient composition and nutritional profile of the diets are presented in Table 1. Results for TMR temperature and microbial counts are summarized in Table 2. The physical characteristics of NW were 1.58% ± 0.3% on upper, 41.8% ± 2.8% on middle, 17.4% ± 1.4% on lower sieves, and 39.3% ± 2.3% in the pan, and WW (mean ± SD) were 6.2% ± 3.7% on upper, 41.9% ± 5.5% on middle, 15.5% ± 4.3% on the lower sieves, and 37.0% ± 4.1% in the pan. The average THI was 68 ± 7, and the average barn temperature was 24°C ± 4°C. There was no treatment effect for TMR temperature (*P* = 0.21), clostridia counts (*P* = 0.58), and total bacteria (*P* = 0.71). However, the NW had higher (*P* = 0.03) yeast counts than WW. A time effect was observed for TMR temperature (*P* < 0.0001) with elevated temperatures as time progressed. Additionally, there was a tendency for a treatment × time point interaction (Figure 1A; *P* = 0.10) where feed temperature tended to be higher for WW (34°C ± 1.56°C, 35°C ± 1.59°C, 35°C ± 1.57°C, 35°C ± 1.49°C) than NW (29°C ± 1.56°C, 29°C ± 1.59°C, 30°C ± 1.57°C, 30°C ± 1.49°C) at 16, 17, 18, and 19 h, respectively. Total bacteria (*P* = 0.04) and yeast (*P* = 0.05) counts increased as hours progressed; meanwhile, clostridia counts (*P* = 0.07) tended to increase during the first 18 h (Figure 1B).

**Table 1:** Ingredient composition and nutritional profile of high-straw prepartum diets^1^

Item	NW	WW
Ingredient, % of DM		
Corn silage^2^	32.13	32.13
Wheat straw	36.48	36.48
Corn gluten feed	4.67	4.67
Water	—	0.03
Rumen-protected lysine^3^	0.17	0.17
Rumen-protected methionine^4^	0.09	0.09
Expeller soybean meal^5^	6.08	6.08
Calcium carbonate	2.21	2.21
Wheat middlings	4.25	4.25
Soybean meal, 47% CP	2.27	2.27
Urea, 46%	0.25	0.25
Anionic mineral supplement^6^	6.24	6.24
Soybean hulls pellet	3.59	3.59
Vitamin premix E20K^7^	0.08	0.08
Magnesium oxide	0.03	0.03
Magnesium sulfate	0.23	0.23
Vitamin and mineral mix prepartum^7^	1.27	1.27
Nutritional profile		
DM, %	60.3 ± 0.04	46.7 ± 1.90
CP, % DM	14.0 ± 0.59	
aNDF,^8^ % DM	45.0 ± 2.39	
ADF, % DM	28.3 ± 3.66	
Ash, % DM	9.60 ± 1.19	
Crude fat, % DM	3.38 ± 0.24	
Starch, % DM	17.2 ± 3.16	

1 High-straw prepartum diets without (NW) or with (WW) water included in the mixture; water represented 23% of the as-fed diet.

2 Corn silage averaged 33.6% DM across diets.

3 AjiPro-L Generation 3 (Ajinomoto Heartland Inc., Chicago, IL).

4 Smartamine M (80% bioavailability providing 1.05 g of Smartamine M/kg of DMI; Adisseo, Alpharetta, GA).

5 SoyPlus (Landus Cooperative, Ames, IA).

6 Biochlor (Arm and Hammer Animal Nutrition, Princeton, NJ).

7 Vitamin and mineral mix prepartum was formulated to contain 28.19% Ca, 3.99% Na, 5.90% Cl, 6.11% Mg, 0.02% K, 0.22% S, 3.70 mg/kg Co, 510.61 mg/kg Cu, 97.47 mg/kg I, 1,257.27 mg/kg Fe, 2,424.62 mg/kg Mn, 27.68 mg/kg Se, 27.68 organic Se, 1,000.74 mg/kg Zn, 720.12 kIU/kg vitamin A, 219.00 kIU/kg vitamin D_3_, and 7,125.16 IU/kg vitamin E.

8 aNDF = neutral detergent fiber assayed with a heat-stable amylase and expressed inclusive of residual ash.

**Table 2:** Least squares means and associated SEM for temperature and microbial counts of dry cow TMR^1^

Variable	Treatment^2^		SEM^4^	*P*-value^3^		
	NW	WW		Trt	Tp	Trt × Tp
Temperature, °C	29.1	30.6	0.76	0.21	<0.0001	0.10
Clostridium, cfu/g	73.1	90.2	25.8	0.58	0.07	0.49
Total bacteria, cfu/g^5^	420.4	448.7	126.2	0.71	0.04	0.38
Yeast, cfu/g^6^	79.2	55.8	34.1	0.03	0.05	0.86

1 Data were recorded for 24 h after mixing the diet.

2 Treatments consisted of a dry cow TMR diet without water (NW) or with water (WW) added to the mixture.

3 Main effects of treatment (Trt), time point (Tp), and their interaction.

4 Greatest value for SEM within treatment.

5 cfu/g × 10^−3^.

6 cfu/g × 10^−2^.

**Figure 1:**
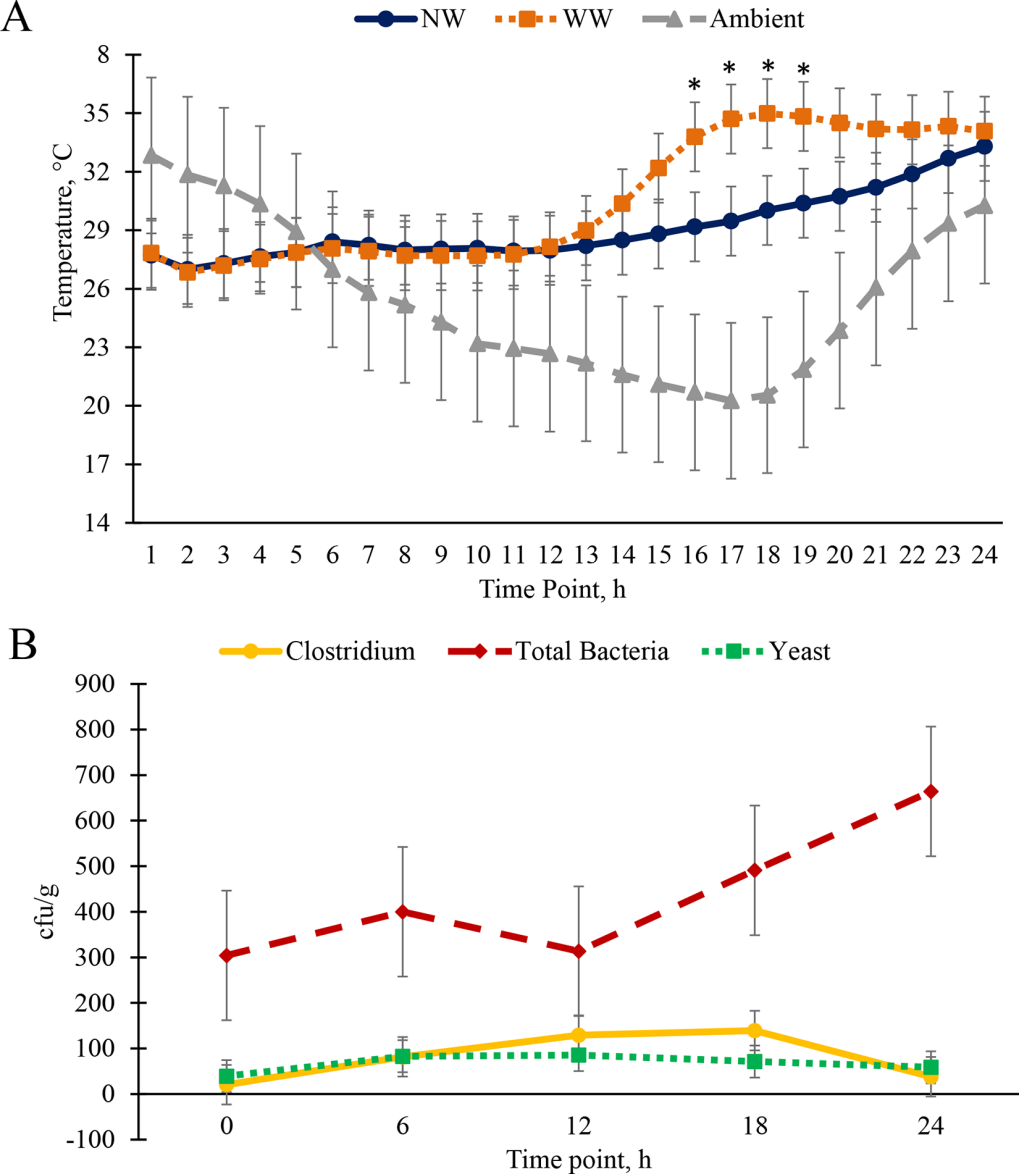
Least squares means (±SEM) for temperature (A) for a high-straw dry cow TMR without (NW) or with the addition of water (WW) over a 24-h period. Time point 1 represents 1 h after feeding (1300 h). *Asterisk indicates a difference between treatments at that time point at *P* ≤ 0.05. (B) Least squares means (±SEM) for microbial populations in a dry cow TMR over a 24-h period; total bacteria counts = (cfu/g × 10^−3^) and yeast counts = (cfu/g × 10^−2^).

Researchers have previously investigated the effect of water addition to dry cow rations with a high straw inclusion rate (Havekes et al., 2020), but to our knowledge the current study is the first to evaluate the effects of water addition on feed temperature and microbial populations of a high-straw dry cow TMR. It has been proposed that adding water to the TMR may help smaller particles adhere to the longer particles, thereby decreasing sorting preferences for the smallest dietary particles (Shaver, 2002; Miller-Cushon and DeVries, 2009). In the current study, the main difference observed in percent distribution of the particles across treatments was in the upper sieve, with NW showing a lower percentage of long particles compared with the WW treatment. Dissimilarly, Havekes et al. (2020) reported no differences in percent distribution of long or medium particles across high-straw dry diets with either 53.5% DM or 45.4% DM but rather a tendency for a lower percentage of fine particles and a higher percentage of short particles for the high-straw dry diet with 45.5% DM. Although cow sorting was not measured in the present study, it is possible that cows fed diets with finely chopped straw may sort against the fine particles (Coon et al., 2018; Havekes et al., 2020). It has been suggested that water inclusion in diets consisting mainly of ensiled forages (50%–60% of total ration DM) may reduce its stability, depending on factors such as the silage's volatile fatty acid profile and the presence of molds and yeasts in the feed (Havekes et al., 2020). As a result, the diet may be more prone to heating and spoilage (Felton and DeVries, 2010), especially within the wettest portion of the ration, typically the long particle fraction (Miller-Cushon and DeVries, 2009; Felton and DeVries, 2010). An increase in TMR temperature was observed in the present study for the WW treatment compared with NW, although the extent of this effect varied across time points, with more pronounced increases in TMR temperature occurring between 16 to 19 h. This is consistent with Felton and DeVries (2010), where it was demonstrated that greater amounts of water added to a lactating diet elevated feed temperature in the hours after feed was delivered to the feed bunk. However, the heating observed in the lactating diet by Felton and DeVries (2010) occurred much earlier, within a 6-h period, compared with the current study. These increases in TMR temperature throughout the day in the feed bunk have been previously noted and linked with TMR spoilage (Kung et al., 1998). In the present study, total bacteria growth was drastically increased after 12 h of feed delivery, which could be associated with the elevated feed temperatures observed as time progressed.

Yeasts can metabolize lactic acid simultaneously and wetter conditions favor the growth of yeasts (Hao et al., 2015). Hao et al. (2015) reported an increased rate of yeast growth in nonfermented TMR with a high moisture level (500 g/kg). Furthermore, the TMR in the aforementioned study was more prone to aerobic spoilage, indicating a relationship between moisture content and yeast activity. Interestingly, our data reported greater yeast counts for NW compared with WW despite the lower moisture content and smaller proportion of long particles. In addition to yeasts, clostridia populations have also been detected in the feed of dairy cows, with the most abundant clostridia species identified as *Paraclostridium bifermentans*, *Clostridium perfringens*, and *C. beijerinckii* (Bretl et al., 2022). Overall, clostridia growth in the TMR tended to increase in the first 18 h after feed delivery regardless of the addition of water into the mixture. In silage, the growth of clostridia is encouraged by a rise in pH resulting from the metabolism of lactic and other acids (Tabacco et al., 2009; Borreani and Tabacco, 2010), which leads to the degradation of both carbohydrates and proteins (Woolford, 1978). Although clostridia are strict anaerobes, their counts can increase during the aerobic deterioration phase (Pahlow et al., 2003), potentially due to the coexistence of aerobic and anaerobic niches within the silage that enable clostridia to benefit from the oxidation of preservative acids by aerobic organisms (Jonsson, 1989). While the pH of the TMR was not assessed in the current study, it is possible that an increase in pH occurred, creating conditions in which yeast activity may have indirectly promoted clostridial growth. From a management perspective, water inclusion may improve ration uniformity but also increase the risk of heating if feed remains exposed, highlighting the importance of timely feeding and careful moisture management on farms. Evening or nighttime feeding is sometimes implemented during warmer weather to ensure cows receive fresh feed during cooler hours with the expectation that cows' activity levels are higher during this time (Aharoni et al., 2005). In the present study, the timing of the TMR being mixed and delivered was in the afternoon during peak temperatures. It is very likely that, if the TMR regardless of the addition of water had been mixed and delivered during cooler morning hours, initial feed temperatures at the start of the 24-h incubation would have been lower, with a slower subsequent rise in temperature throughout the day. In conclusion, adding water to a high-straw dry cow TMR increased feed temperature after 16 h of preparation, whereas yeast counts were lower over time in the diet with added water. Total bacterial and clostridial counts were not affected by water addition but increased over time. Because feeding energy-dense diets during the close-up period remains common practice, additional studies are warranted to understand whether similar effects are observed with different diet compositions.
